# Pharmaceutical Values of Calycosin: One Type of Flavonoid Isolated from *Astragalus*

**DOI:** 10.1155/2021/9952578

**Published:** 2021-05-06

**Authors:** Guowei Gong, Yuzhong Zheng, Yang Yang, Yixuan Sui, Zhen Wen

**Affiliations:** ^1^Department of Bioengineering, Zunyi Medical University, Zhuhai Campus, Zhuhai, Guangdong 519041, China; ^2^Guangdong Key Laboratory for Functional Substances in Medicinal Edible Resources and Healthcare Products, School of Life Sciences and Food Engineering, Hanshan Normal University, Chaozhou, Guangdong 521041, China; ^3^Department of Neuroscience, City University of Hong Kong, Hong Kong 999077, China; ^4^College of Chemistry and Environmental Engineering, Shenzhen University, Shenzhen 518060, China

## Abstract

*Astragalus* is a popular *Materia Medica* in China, and it could be applied in the treatment of various diseases. It contains a variety of chemically active ingredients, such as saponins, flavonoids, and polysaccharides. Plant-derived bioactive chemicals are considered natural, safe, and beneficial. Among the infinite plant-identified and isolated molecules, flavonoids have been reported to have positive effects on human health. Calycosin is the most important active flavonoid substance identified predominantly within this medicinal plant. In recent years, calycosin has been reported to have anticancer, antioxidative, immune-modulatory, and estrogenic-like properties. This review collected recent relevant literatures on calycosin and summarized its potential pharmaceutical properties and working mechanism involved, which provided solid basis for future clinical research.

## 1. Introduction

The development of traditional Chinese medicine (TCM) has a history of thousands of years, and it has accumulated myriad medical experience and summarized pharmacological effects of *Materia Medica* playing a pivotal role in modernization of TCM [1, 2]. With the development of modern medicine, the pharmaceutical properties of raw herbal extract can no longer satisfy today's sophisticated biomedical research. The bioactive molecules selected and isolated from plant are more suitable as potential medicine [3, 4]. In fact, plant-derived chemicals are associated with drug development, such as Taxol isolated from *Taxus chinensis* and camptothecin identified and enriched from *Camptotheca acuminata.*

*Astragalus membranaceus*, Huangqi in Chinese, as a classic traditional herbal medicine, is commonly used in a variety of traditional Chinese medicine prescriptions [5, 6]. The major pharmaceutical functions of this *Materia Medica* are boosting immune and hematopoietic systems [7, 8]. Previous studies have reported that a plethora of flavonoids have been identified and isolated from *Astragalus.* Flavonoids are classified as polyphenolic compounds and they are ubiquitously enriched in the plant kingdom [9, 10]. It is estimated that over 4000 flavonoids have been reported and identified, and they could be clustered into 8 subclasses, that is, flavanole, flavanonole, chalkone, anthocyanidine, aurone, flavone, flavanone, and isoflavone [9, 10]. Calycosin is the most enriched isoflavone found abundantly in *Astragalus.* This molecule has gained attention for its myriad medical functions both *in vitro* and *in vivo* [2, 5, 6, 11]. Hence, this review emphasizes the effects of calycosin on anticancer, antioxidative, immune-modulatory, and estrogenic-like properties.

## 2. Pharmacological Activities of Calycosin

### 2.1. Anticancer Functions

Breast cancer is one of the most common cancers threatening women globally and it accounts for approximately 15% of female cancer-related deaths in the United States [12, 13]. Human breast cancer is classified into estrogen receptor-positive (ER+) and estrogen receptor-negative (ER−) subtypes. Tian et al. have reported that calycosin was able to inhibit both ER− and ER+ breast cancer cell proliferation in a dose-dependent manner and the inhibitory effects were associated with noncoding RNA WDR7-7 expression level by inducing G-protein coupled estrogen receptor 30 (GPR30) and RASD1 via Erk1/2 and Akt transduction pathway [14, 15]. The apoptosis-related protein, cleaved caspase 3/9, and Bax were significantly stimulated under the treatment of calycosin in ER + cancer cell type MCF-7 [14]. Li group published similar data and confirmed that calycosin at 150 *μ*M was capable of blocking MCF-7 and T47D cells migration and invasion by wound healing and Transwell assays [16]. Interestingly, calycosin at 2 *μ*mol/L already triggered MCF-7 cell apoptosis by flow cytometry analysis [17]. Additionally, treatment of calycosin could downregulate forkhead box P3, vascular endothelial growth factor (VEGF), and matrix metalloproteinase 9 (MMP9) in MCF-7 and T47D [17]. Furthermore, Chen group (2014) confirmed that calycosin induced ER+ MCF-7 cell apoptosis via the blocking insulin-like growth factor 1 receptor (IGF-1R) pathway after 48-hour treatment [18]. On the other hand, Wu et al. (2019) found that the application of calycosin decreased invasive and migratory effects in ER-breast cancer MDA-MB231 cells by suppressing Rab27B, *ß*-catenin, and VEGF levels. More importantly, the inhibitory activities under the challenge of calycosin were recovered by the overexpression of Rab27B [19].

Colorectal cancer has a high mortality rate, which is also named as bowel cancer, colon cancer, or rectal cancer, claiming at least 500 thousand lives every year globally [20, 21]. Colorectal cancer is the third highest incidence of all cancers worldwide. The early symptoms of colorectal cancer are hard to detect, and the terminal stage of colorectal cancer is barely treated due to lack of effective biomarkers for clinical screening [22]. The study found that the potential targets of calycosin on colorectal cancer were ER*α*, ER*β*, ATP-binding cassette subfamily G member 2, breast cancer type 1 susceptibility protein, CYP19A1, and epidermal growth factor receptor (EGFR) [22]. Therefore, these targets could be used as monitor for colorectal cancer treatment. Besides, the *in vitro* and *in vivo* against colorectal properties of calycosin have been widely documented [23–25]. Zhao et al. have published that calycosin suppressed colorectal cancer cell line SW480 dose-dependently by Hoechst 33258 assay [25]. Furthermore, the xenograft tumor size in nude mice was decreased by the calycosin treatment [25]. Impressively, calycosin significantly enhanced autophagy specific protein expressions, that is, Beclin-1 and LC-3II, after 48-hour incubation in cultured HT-29 cells [26]. However, cotreatment of HT-29 with IGF-1 could recover calycosin-induced cell autophagy. Wang found that calycosin inhibited colorectal cancer proliferation and migration by enhancing BATF2 to target plasminogen activator inhibitor-1 [27]. Moreover, this molecule was able to abolish transforming growth factor *ß*- (TGF-*β*-) induced epithelial-to-mesenchymal transition via altering Wnt mechanism [27]. In addition, calycosin robustly restricted HCT-116 cells viability and invasiveness by enhancing ER*β* and phosphatase and tensin homolog (PTEN) expressions [28].

Osteosarcoma is the most common malignant bone tumor with potential for invasion and metastasis; however, the current chemotherapy for osteosarcoma is not yet perfect [29, 30]. Calycosin is evidenced to induce MG-63 apoptosis, reduce cell proliferation, and decrease matrix metalloproteinase 2 (MMP2) and proliferating cell nuclear antigen expression after 48-hour incubation [31]. In tumor-bearing nude mice study, the tumor size and weight were reduced in calycosin-treated group [31]. Protein expression levels of I*κ*B*α* and interleukin-6 (IL-6) were attenuated after calycosin interference for 3 weeks [31]. The data was in line with Wang et al.'s work published in 2018; they found that calycosin suppressed PI3K/AKT/mTOR pathway. In the MG-63 xenografts nude mice, calycosin inhibited tumor growth and also regulated phosphorylations of PI3K/Akt [31–33]. Hence, Table 1 summarizes the anticancer functions of calycosin.

### 2.2. Antioxidative Properties

Oxidative stress is a phenomenon triggered by the excessive production and accumulation of reactive oxygen species (ROS) in cells and finally leads to dysfunction of tissues [34, 35]. Calycosin has been evidenced to protect doxorubicin-induced oxidative stress in cultured cardiomyocyte by inhibiting ROS generation via enhancing antioxidant enzymatic activities, that is, glutathione peroxidase, catalase, and superoxide dismutase (SOD) (Table 2) [36]. Moreover, the levels of sirtuin 1-NOD-like receptor protein 3 and related proteins were elevated after calycosin was incubated for 24 hours both *in vitro* and *in vivo* [36]. Liu found that calycosin could also attenuate H_2_O_2_-induced H9C2 cell apoptosis rate in a dose-dependent manner [38]. Pretreatment with ER antagonist, ICI 182,780, negated the protective effect of calycosin against H_2_O_2_-induced apoptosis [37, 38]. Elsherbiny et al. (2020) have reported that calycosin showed potential effects on type 2 diabetes mellitus treatment after 4-week administration [40]. The contents of IL33/ST2 mRNA were enhanced and levels of p65, tumor necrosis factor *α* (TNF-*α*), interleukin-1*β* (IL-1*β*) and TGF-*β* were downregulated in calycosin treatment mice (Table 2) [39, 40]. Interestingly, calycosin reduced oxidative stress in intracerebral hemorrhage mouse model by stimulating Nrf-2 protein expression [42]. Oral administration of calycosin at 25 or 50 mg/kg/day was able to enhance amylase and lipase levels in serum in acute pancreatitis rat model [41]. The cytokine levels after calycosin treatment were mitigated [41]. Additionally, calycosin was able to decrease cerulein-induced pancreatic edema, inhibiting myeloperoxidase activity and stimulating SOD activity [41]. Studies have shown that calycosin can extend the lifespan of *C. elegans*, and this extension is related to the antioxidant capacity by enhancing stress resistance capacity and reducing the accumulation of ROS [43]. Lu group (2017) published that calycosin required insulin signaling involvement to promote lifespan extension [43]. On the other hand, they observed that calycosin can enhance the nuclear translocation of the core transcription factor DAF-16/FOXO, rather than the conservative stress response transcription factor SKN-1/Nrf-2 [43].

### 2.3. Anti-Inflammatory Functions

The anti-inflammatory properties of calycosin were widely documented on lipopolysaccharide- (LPS-) induced RAW 264.7 cells [41, 44]. Calycosin significantly attenuated nitric oxide (NO), prostaglandin E2 (PGE2), TNF-*α*, IL-1*β*, and IL-6 releases, and the anti-inflammatory properties had been confirmed by NF-*κ*B and MAPK signal pathways [44]. The effective dosage was from 30 nM to 5 *μ*M, and the inhibitory function was dose-dependent. Besides, calycosin could also diminish inflammatory cell markers CD68 and F4/80 mRNA levels in a dose-dependent manner [45].

Calycosin was reported to possess renal protective functions in high-fat diet-induced type 2 diabetes mellitus rat model by altering SOD and TGF-*β* content in renal tissues as compared to the sham group [40]. Zhang et al. published similar data and reported that this molecule was able to effectively alleviate kidney injury in diabetic kidneys of db/db mice after treatment for 28 days (Table 3) [50]. The serum contents of inflammatory cytokines were reduced via suppressing I*κ*B*α* and NF-*κ*B p65 [50]. Additionally, fed glucose level in db/db obese mice was declined after calycosin administration which was proposed to be related to the anti-inflammatory effects [45]. Reduced serum triglyceride levels, alleviated insulin resistance, and glucose intolerance were observed in calycosin-treated mice compared with the vehicle-treated controls [45].

Xu et al. found that calycosin was able to relieve advanced glycation end products- (AGE-) induced inflammation both *in vitro* and *in vivo* [49]. AGEs act as the central role in vasculitis development by recruiting the receptor for AGE overexpression (Table 3) [49]. Calycosin was able to diminish vasculitis development by downregulating the AGEs-induced overexpression of receptor for advanced glycation end products (RAGE) and proinflammatory cytokines in both rat and HUVECs [46]. ERK1/2 and NF-*κ*B pathways were involved and evidenced by Kim et al. and Cheng et al. after calycosin presence for 4 hours [47, 48].

### 2.4. Estrogenic-Like Properties

#### 2.4.1. Osteogenic Functions of Calycosin

Women suffering from menopause have higher risk of getting osteoporosis. The role of calycosin in preventing osteoporosis is widely reported [51, 52]. The proliferation and differentiation capacities of MG-63 were determined with and without calycosin presence [52]. From the results, calycosin was able to stimulate osteoblast differentiation dose-dependently [52]. The data was further confirmed in the rat primary cultured osteoblast. Data implied that the alkaline phosphatase (ALP) level and Runx2 were significantly enhanced after exposure of calycosin for 48 hours [52, 53]. Fang group found that calycosin could also modulate GSK-3*β* pathway for stimulating osteoblast differentiation which was further confirmed by its specific inhibitor GSK1904529A after revealing ALP, Col1a1, and Runx2 expression levels [54]. Bone mineral density was robustly enhanced in ovariectomized (OVX) rats after administration of calycosin for 12 weeks [51]. Elevated serum level of ALP and declined tartrate-resistant acid phosphatase (TRAP) level were observed [51]. Calycosin could also stimulate osteoprotegerin transcriptional and translational activities and downregulate RANKL expression level in calycosin group as compared with OVX group, and these alternations were proposed to be correlated with MAPK pathway. On the other hand, calycosin was able to abolish RANKL-induced osteoclast formation from primary bone marrow macrophages dose-dependently after 24-hour incubation [55]. Therefore, calycosin may be useful as a therapeutic agent for bone loss-associated diseases.

#### 2.4.2. Hematopoietic Functions of Calycosin

One symptom of estrogen deficiency is anemia [7]. Several lines of evidence suggested that calycosin could stimulate the expression of erythropoietin (EPO), the central regulator of red blood cell mass, in cultured human embryonic kidney fibroblasts (HEK293T) after exposure of calycosin for 24 hours [7, 8, 11, 35]. The calycosin-induced EPO expression was mediated by HIF-1*α* from western blotting results [56]. The *in vivo* experiments showed that calycosin could enhance the number of RBCs, WBCs, PLTs, and content of Hb in peripheral blood and the area of bone marrow hematopoietic tissue [57]. The serum contents of thrombopoietin, EPO, granulocyte-macrophage colony stimulating factor, colony of CFU-GM, CFU-MK, CFU-E, and BFU-E were also enriched after calycosin treatment [57]. The animal experiments showed that this agent reduced G0/G1 cells and increased G2/M cells in hematopoietic stem cells.

### 2.5. Neuroprotective Functions

The neuroprotective functions of calycosin were determined both *in vitro* and *in vivo*. Administered with different concentration of calycosin from 7.5 mg/kg/day to 30 mg/kg/day, reduced malondialdehyde (MDA) and ROS contents were observed in calycosin-treated ischemia reperfusion rats [58]. However, the SOD activity was induced in the calycosin-treated ischemia reperfusion rats [58]. Calycosin could also stimulate ER*β*, miR-374, and Bcl-2 protein expression levels in middle cerebral artery occlusion rats from western blotting data [59]. Similar protective effects of calycosin were evidenced *in vitro* by utilizing PC12 neuronal cell line with pretreatment of l-glutamate or xanthine (XA)/xanthine oxidase (XO) [60, 61]. Calycosin showed potential neuroprotective functions by blocking XA/XO-induced cell apoptosis at ∼50% and the EC50 of 0.05 mg/L and an IC50 of approximately 50 mg/L [60–62]. Interestingly, calycosin shows promising therapeutic value on Alzheimer's disease in APP/PS1 transgenic mice [63]. Intraperitoneally injected calycosin, the diminished hippocampal beta amyloid, Tau protein, IL-1*β*, TNF-*α*, acetylcholinesterase, and MDA levels were observed and the inhibitory effects were in a dose-dependent manner [63]. The maximal blockage concentration was revealed at 40 mg/kg/day. Yang found that calycosin could also mitigate Parkinson's disease in 1-methyl-4-phenyl-1,2,3,6-tetrahydropyridine- (MPTP-) induced Parkinson's disease mice [64]. From the results, Yang et al. confirmed that calycosin treatment mitigated the behavioral dysfunctions and inflammatory responses in MPTP-induced PD mice via NF-*κ*B/MAPK pathways [64].

## 3. Pharmacokinetics of Calycosin

Because of hydroxyl groups found within the chemical structure of calycosin, they are metabolized to glucuronide by phase II metabolic enzymes such as UDP-glucuronosyltransferases from the intestine and liver after oral administration [65]. In addition to the role of metabolism, absorption, hydrolysis, efflux, and intestinal circulation in the intestinal tract also participate in the disposal of calycosin in the body, affecting their systemic and local bioavailability [66].

Studies have shown that, after oral administration of *Astragalus* water extract, the enriched content of calycosin-7-O-*β*-glucoside is detected in plasma, indicating that calycosin-7-O-*β*-glucoside can enter the intestinal cells in a prototype form and be metabolized [66, 67]. In the study, it was found that, after administration of calycosin by oral gavage, calycosin-7-O-*β*-glucoside can be detected in the plasma, which shows that calycosin-7-O-*β*-glucoside can penetrate the cell membrane in a prototype form and undergo further metabolism [68]. This indicates that calycosin-7-O-*β*-glucoside may directly pass through the cell membrane in a prototype form without hydrolysis [69]. Using Caco-2 cells to study the absorption and transport characteristics of calycosin and its glucoside, it was found that calycosin and calycosin-7-O-*β*-glucoside are mainly absorbed in the form of passive diffusion, and the absorption process will not be affected by inhibitors of MATEs such as P-gp and MRP2 [69, 70]. Calycosin can be metabolized in human liver microsomes to generate two glucuronides. UGT1A1 and UGT1A9 are the major metabolic enzyme subtypes that generate these two glucuronides, respectively [71]. On the other hand, after oral administration of calycosin, the calculated bioavailability of calycosin-7-O-*β*-glucoside was only 0.304%, indicating that hydrolyzing is an important process of metabolism *in vivo* [72, 73]. However, glucoside hydrolase is solely present in the intestine and liver of rats. In order to confirm the hydrolysis site of calycosin-7-O-*β*-glucoside, the pharmacokinetics of calycosin-7-O-*β*-glucoside injection in rats were investigated because the drug was directly absorbed by the hepatic portal vein after intraperitoneal injection [74]. The drug will not be processed through the intestine, so as to exclude the effect of the intestine on the calycosin-7-O-*β*-glucoside treatment in the body [75]. The results show that the drug time curve of calycosin-7-O-*β*-glucoside and its metabolites is completely different from that of calycosin-7-O-*β*-glucoside after oral administration.

## 4. Chemical Interactions

As the main bioactive molecule isolated from Astragali Radix, the pharmacological activity of calycosin is not performed alone but by the joint action of multiple chemical substances. Cotreatment of calycosin with other biochemicals identified from Astragali Radix, that is, formononetin, ononin and astragaloside, showed effective therapeutic functions as compared to single compound. The study found that the expression levels of drug-metabolizing enzymes, such as CYP3A4, CYP2B6, CYP2E1, UGT1A, and efflux transporters, that is, P-gp, MRP2, BCRP, and MRP3, were increased in a dose-dependent manner in the drug combination group [76]. Zheng et al. found that cotreatment of calycosin with formononetin stimulated EPO expression in a dose- and concentration-dependent manner in cultured HEK 293 cells. The hematopoietic functions of these combinations were even stronger than the positive control [77, 78]. Furthermore, Zhang et al. reported that flavonoid combination containing formononetin and calycosin at weight ratio of 1 : 5 showed the best hematological functions on anemic rat after drug treatment [79].

The research of calycosin in modern medicine is no longer confined to a single compound or Astragali Radix. More and more scientists have discovered the combination of multiple substances enjoying a broad spectrum in disease treatment by “Fu Fang.” Astragali Radix and Angelicae Sinensis Radix are usually combined together clinically [35, 52]. Cotreatment of calycosin and Astragali Sinensis Radix-derived ferulic acid protected bleomycin-induced pulmonary fibrosis in rats, and this action was believed via blocking NOX4 expression [80]. Furthermore, combination of calycosin and ferulic acid showed better immune-modulatory pharmaceutical activities in Raw 264.7 and inducing blood vessels in HUVECs and Zebra fish [81–85]. Administration of calycosin and ferulic acid attenuates cytokine and inflammatory mediators' releases in atopic dermatitis-like mouse [85].

## 5. Conclusion

Calycosin serves as a common dietary flavonoid and is consumed in daily cuisine and/or TCM decoction. Additionally, there are myriad of formulae containing calycosin at different dosage forms either alone or in combination with other bioactive molecules in market. The pharmaceutical functions of calycosin on anticancer, antioxidative, immune-modulatory, and estrogenic-like properties were summarized. We believe the potential pharmaceutical value of calycosin is still behind the veil, and which motivating us to discover more in the future. The *in vitro* and *in vivo* pharmaceutical functions do not directly translate into the clinic because of bioavailability and biotransformation influenced by gut microbiota. Considering various compositions of microbiota between individuals, the fluctuating process of bioavailability and biotransformation mediated by gut microbiota could have a consequential effect of calycosin and its metabolites in plasma, finally leading to diverse clinic functions. Hence, gut microbiota-induced bioavailability and biotransformation of calycosin and its metabolites should be taken into consideration before clinical application.

## Figures and Tables

**Table 1:** Anticancer functions of calycosin

Biomarkers	Model	Reference
Cleaved caspase 3/9 upregulation; enhanced Bax/Bcl-2 ratio	MCF-7 cells; MDA-MB231 cells	Tian et al. [14]; Tian et al. [15]
Reduced cell migration and invasion; enhanced apoptosis rate via blocking IGF-1R pathway; downregulated VEGF and MMP	MCF-7 cells; T47D cells	Li et al. [16]; Chen et al. [17]; Chen et al. [18]
Downregulation of Rab27B and *β*-catenin	MDA-MB-231 cells	Wu et al. [19]
Abnormal expression levels of ER*α*, ER*β*, ATP-binding cassette subfamily G member 2, breast cancer type 1 susceptibility protein, p450, and EGFR	OMIM data	Huang et al. [22]
Colorectal cancer proliferation	SW480 cells	Zhao et al. [25]
Beclin-1 and LC-3II overexpression	HT-29 cells	EI-kott et al. [26]
Upregulation of ER*β* and PTEN	HCT-116 cells	Chen et al. [28]
Induced osteosarcoma apoptosis and decrease MMP2	MG-63 cells	Qiu et al. [31]
Reduced tumor size and PI3K/Akt phosphorylation	Nude mice	Wang et al. [32]; Qiu et al. [31]; Tian et al. [33]

**Table 2:** Antioxidative functions.

Biomarkers	Model	Reference
Enhance glutathione peroxidase, catalase, and superoxide dismutase enzymatic activities	H9C2 cells and male Kunming mice	Zhai et al. [36]
Reduced H_2_O_2_-induced cell apoptosis rate	H9C2	Chen et al. [37]; Liu et al. [38]
Downregulation of cytokine levels and IL33/ST2 mRNA level	Type 2 diabetes mellitus rat model	Wang & Zhao [39]; EIsherbiny et al. [40]
Stimulating Nrf2 expression and SOD levels	Intracerebral hemorrhage mouse	Ma et al. [41]; Chen et al. [42]
Prolong lifespan and stimulate SOD levels	*C. elegans*	Lu et al. [43]

**Table 3:** Anti-inflammatory functions.

Biomarkers	Model	Reference
Declined NO, PGE2, TNF-*α*, and other cytokine release activities	RAW 264.7 cells	Dong et al. [44]; Ma et al. [41]
Reduced CD68 and F4/80 mRNA levels	Raw 264.7	Hoo et al. [45]
Enhanced SOD and TGF-*β* contents	Type 2 diabetes mellitus rat model	EIsherbiny et al. [40]
Reduced I*κ*B*α* and NF-*κ*B p65 protein translational levels and alleviated insulin resistance	db/db obese mice	Hoo et al. [45]
Alleviated inflammatory responses and cytokine levels via attenuating Erk1/2 and NF-*κ*B pathways	Hepatocyte cell line; HUVEC	Figarola et al. [46]; Kim et al. [47]; Cheng et al. [48]; Xu et al. [49]

## Data Availability

The data used in this paper are available upon request.

## Abbreviations

ALP: Alkaline phosphatase
EGFR: Epidermal growth factor receptor
EPO: Erythropoietin
ER−: Estrogen receptor-negative
ER+: Estrogen receptor-positive
GPR30: G-protein coupled estrogen receptor 30
IGF-1R: Insulin-like growth factor 1 receptor
IL-1*β*: Interleukin-1*β*
IL-6: Interleukin-6
LPS: Lipopolysaccharide
MMP2: Matrix metalloproteinase 2
MMP9: Matrix metalloproteinase 9
NO: Nitric oxide
OVX: Ovariectomized
PGE2: Prostaglandin E2
PTEN: Phosphatase and tensin homolog
RAGE: Receptor for advanced glycation end products
ROS: Reactive oxygen species
SOD: Superoxide dismutase
TCM: Traditional Chinese medicine
TGF-*β*: Transforming growth factor *ß*
TNF-*α*: Tumor necrosis factor *α*
TRAP: Tartrate-resistant acid phosphatase
VEGF: Vascular endothelial growth factor
MDA: Malondialdehyde
XA/XO: Xanthine/xanthine oxidase
MPTP: 1-Methyl-4-phenyl-1,2,3,6-tetrahydropyridine.
